# Supplementary material for: Agreement between Type 2 Diabetes Risk Scales in a Caucasian Population: A Systematic Review and Report
Source: J Clin Med. 2020 May 20;9(5):1546. doi: 10.3390/jcm9051546 (PMC7290893; doi:10.3390/jcm9051546)
Supplement: Supplementary file 1 [file jcm-09-01546-s001.pdf]

Supplementary material: Table S1. Distribution of people at high risk using DETECT-2, DDRS, FINDRISC, EGATS, NUDS, Hisayama, ITD and ARIC score.

		DETECT-2		DDRS		Cambridge		FINDRISC		EGATS		NUDS		Hisayama		AUSDRISK		ITD		ARIC	
**DETECT-2**		*nHR*	*HR*	*nHR*	*HR*	*nHR*	*HR*	*nHR*	*HR*	*nHR*	*HR*	*nHR*	*HR*	*nHR*	*HR*	*nHR*	*HR*	*nHR*	*HR*	*nHR*	*HR*
	nHR	NA	NA	47,443	3,227	45,344	5,308	45,780	4,920	40,569	10,101	30,720	19,967	39,602	11,038	42,396	8,245	50,195	483	50,340	360
	HR	NA	NA	3,734	4,603	1,972	6,354	1,002	7,340	553	7,788	484	7,858	1,128	7,214	899	7,356	7,008	1,325	4,953	3,389
	Kappa			*0.501*		*0.564*		*0.654*		*0.497*		*0.277*		*0.432*		*0.531*		*0.222*		*0.518*	
**DDRS**		*nHR*	*HR*	*nHR*	*HR*	*nHR*	*HR*	*nHR*	*HR*	*nHR*	*HR*	*nHR*	*HR*	*nHR*	*HR*	*nHR*	*HR*	*nHR*	*HR*	*nHR*	*HR*
	nHR	47,443	3,734	NA	NA	46,235	4,880	44,910	6,267	40,603	10,543	31,198	19,977	38,787	12,345	41,384	9,742	50,845	326	49,507	1,670
	HR	3,227	4,603	NA	NA	1,055	6,773	1,851	5,979	503	7,327	0	7,830	1,941	5,889	1,891	5,844	6,345	1,482	5,755	2,075
	Kappa	*0.501*				*0.638*		*0.518*		*0.473*		*0.293*		*0.327*		*0.395*		*0.271*		*0.298*	
**Cambridge**		*nHR*	*HR*	*nHR*	*HR*	*nHR*	*HR*	*nHR*	*HR*	*nHR*	*HR*	*nHR*	*HR*	*nHR*	*HR*	*nHR*	*HR*	*nHR*	*HR*	*nHR*	*HR*
	nHR	45,344	1,972	46,235	1,055	NA	NA	42,789	4,517	40,085	7,201	30,914	16,389	38,799	8,457	40,276	6,991	47,041	257	46,354	8,881
	HR	5,308	6,354	4,880	6,773	NA	NA	3,961	7,701	1,036	10,625	290	11,372	1,931	9,731	3,001	8,565	10,104	1,545	962	2,781
	Kappa	*0.564*		*0.638*				*0.555*		*0.633*		*0.414*		*0.541*		*0.524*		*0.187*		*0.293*	
**FINDRISC**		*nHR*	*HR*	*nHR*	*HR*	*nHR*	*HR*	*nHR*	*HR*	*nHR*	*HR*	*nHR*	*HR*	*nHR*	*HR*	*nHR*	*HR*	*nHR*	*HR*	*nHR*	*HR*
	nHR	45,780	1,002	44,910	1,851	42,789	3,961	NA	NA	40,122	6,630	30,409	16,363	37,772	8,956	41,231	5,497	46,614	152	46,397	385
	HR	4,920	7,340	6,267	5,979	4,527	7,701	NA	NA	1,000	11,259	795	11,462	2,958	9,296	2,064	10,104	10,589	1,656	8,896	3,364
	Kappa	*0.654*		*0.518*		*0.555*				*0.664*		*0.399*		*0.480*		*0.645*		*0.193*		*0.358*	
**EGATS**		*nHR*	*HR*	*nHR*	*HR*	*nHR*	*HR*	*nHR*	*HR*	*nHR*	*HR*	*nHR*	*HR*	*nHR*	*HR*	*nHR*	*HR*	*nHR*	*HR*	*nHR*	*HR*
	nHR	40,569	553	40,603	503	40,085	1,036	40,122	1,000	NA	NA	29,827	11,286	36,170	4,895	38,733	2,351	40,974	138	40,919	203
	HR	10,101	7,788	10,543	7,327	7,201	10,625	6,630	11,259	NA	NA	1,353	16,532	40,701	18,250	4,532	13,249	16,198	1,670	14,343	3,546
	Kappa	*0.497*		*0.473*		*0.633*		*0.664*				*0.562*		*0.624*		*0.713*		*0.121*		*0.249*	
**NUDS**		*nHR*	*HR*	*nHR*	*HR*	*nHR*	*HR*	*nHR*	*HR*	*nHR*	*HR*	*nHR*	*HR*	*nHR*	*HR*	*nHR*	*HR*	*nHR*	*HR*	*nHR*	*HR*
	nHR	30,720	484	31,198	0	30,914	290	30,409	795	29,827	1,353	NA	NA	29,307	1,855	29,278	1,911	31,183	16	30,946	24,334

	HR	19,967	7,858	19,977	7,830	16,389	11,372	16,363	11,462	11,286	16,532	NA	NA	11,422	16,396	14,004	13,690	26,007	1,792	258	3,491
Kappa		*0.277*		*0.293*		*0.414*		*0.399*		*0.562*				*0.540*		*0.444*		*0.067*		*0.123*	
**Hisayama**		*nHR*	*HR*	*nHR*	*HR*	*nHR*	*HR*	*nHR*	*HR*	*nHR*	*HR*	*nHR*	*HR*	*nHR*	*HR*	*nHR*	*HR*	*nHR*	*HR*	*nHR*	*HR*
	nHR	39,602	1,128	38,787	1,941	38,799	1,931	37,772	2,958	36,170	4,531	29,307	11,422	NA	NA	38,579	2,063	40,515	209	40,505	225
	HR	11,038	7,214	12,345	5,889	8,457	9,731	8,956	9,296	4,895	13,355	1,855	16,396	NA	NA	4,656	13,538	16,630	1,599	14,728	3,524
Kappa		*0.432*		*0.327*		*0.541*		*0.480*		*0.624*		*0.540*				*0.722*		*0.110*		*0.240*	
**AUSDRISK**		*nHR*	*HR*	*nHR*	*HR*	*nHR*	*HR*	*nHR*	*HR*	*nHR*	*HR*	*nHR*	*HR*	*nHR*	*HR*	*nHR*	*HR*	*nHR*	*HR*	*nHR*	*HR*
	nHR	42,396	899	41,384	1,891	40,276	3,001	41,231	2,064	38,733	4,532	29,278	14,004	38,579	4,656	NA	NA	43,067	216	43,290	5
	HR	8,245	7,356	9,742	5,844	6,991	8,565	5,497	10,104	2,351	13,249	1,911	13,690	2,063	13,538	NA	NA	14,016	1,566	11,869	3,732
Kappa		*0.531*		*0.395*		*0.524*		*0.645*		*0.713*		*0.444*		*0.722*				*0.133*		*0.316*	
**ITD**		*nHR*	*HR*	*nHR*	*HR*	*nHR*	*HR*	*nHR*	*HR*	*nHR*	*HR*	*nHR*	*HR*	*nHR*	*HR*	*nHR*	*HR*	*nHR*	*HR*	*nHR*	*HR*
	nHR	50,195	7,008	50,845	6,345	47,041	10,104	46,614	10,589	40,974	16,198	31,183	26,007	40,515	16,630	43,067	14,016	NA	NA	54,321	2,882
	HR	483	1,325	326	1,482	257	1,545	152	1,656	138	1,670	16	1,792	209	1,599	216	1,566	NA	NA	945	863
Kappa		*0.222*		*0.271*		*0.187*		*0.193*		*0.121*		*0.067*		*0.110*		*0.133*				*0.281*	
**ARIC**		*nHR*	*HR*	*nHR*	*HR*	*nHR*	*HR*	*nHR*	*HR*	*nHR*	*HR*	*nHR*	*HR*	*nHR*	*HR*	*nHR*	*HR*	*nHR*	*HR*	*nHR*	*HR*
	nHR	50,340	4,953	49,507	5,755	46,354	8,881	46,397	8,896	40,919	14,343	30,946	24,334	40,505	14,728	43,290	11,869	54,321	945	NA	NA
	HR	360	3,389	1,670	2,075	962	2,781	385	3,364	203	346	258	3,491	225	3,524	5	3,732	2,882	863	NA	NA
Kappa		*0.518*		*0.298*		*0.293*		*0.358*		*0.249*		*0.123*		*0.240*		*0.316*		*0.281*			

DETECT-2: Diabetes and Impaired Glucose Tolerance; DDRS: Danish diabetes Risk score; Cambridge: Cambridge Risk Score; FIDRISC: FINnish Diabetes RIsk SCore risk; EGATS: Electric Generating Authority of Thailand Study; NUDS, National Urban Diabetes Survey; Hisayama: Hisayama study; AUSDRISK: AUStralian Type 2 Diabetes RISK; ITD: Instrument for type 2 diabetes; ARIC: Atherosclerosis Risk in Communities. NA: Non-Applicable; HR: high risk; nHR: non-high ris
